# Supplementary material for: Simultaneous Synthesis of Vitamins D2, D4, D5, D6, and D7 from Commercially Available Phytosterol, β-Sitosterol, and Identification of Each Vitamin D by HSQC NMR
Source: Metabolites. 2019 Jun 6;9(6):107. doi: 10.3390/metabo9060107 (PMC6630616; doi:10.3390/metabo9060107)
Supplement: Supplementary file 1 [file metabolites-09-00107-s001.pdf]

Supplementary Materials

Simultaneous Synthesis of Vitamins D_2, D_4, D_5, D_6, and D_7 from Commercially Available Phytosterol, β-Sitosterol, and Identification of Each Vitamin D by HSQC NMR

Shiro Komba *, Eiichi Kotake-Nara and Wakako Tsuzuki

Food Component Analysis Unit, Food Research Institute, National Agriculture and Food Research Organization, 2-1-12, Kannondai, Tsukuba, Ibaraki 305-8642, Japan; ekotake@affrc.go.jp (E.K.-N.); wakako@affrc.go.jp (W.T.)
*Correspondence: skomba@affrc.go.jp; Tel.: +81-29-838-7298

Figure S1. Synthesis of vitamin D_3. S2

The experimental details on the synthesis of vitamin D_3 (**13**). S2–S3

NMR spectra of vitamin D_2 (**9**) S4–S7

NMR spectra of vitamin D_3 (**13**) S8–S11

NMR spectra of vitamin D_4 (**10**) S12–S15

NMR spectra of vitamin D_5 (**6**) S16–S19

NMR spectra of vitamin D_6 (**8**) S20–S23

NMR spectra of vitamin D_7 (**7**) S24–S27

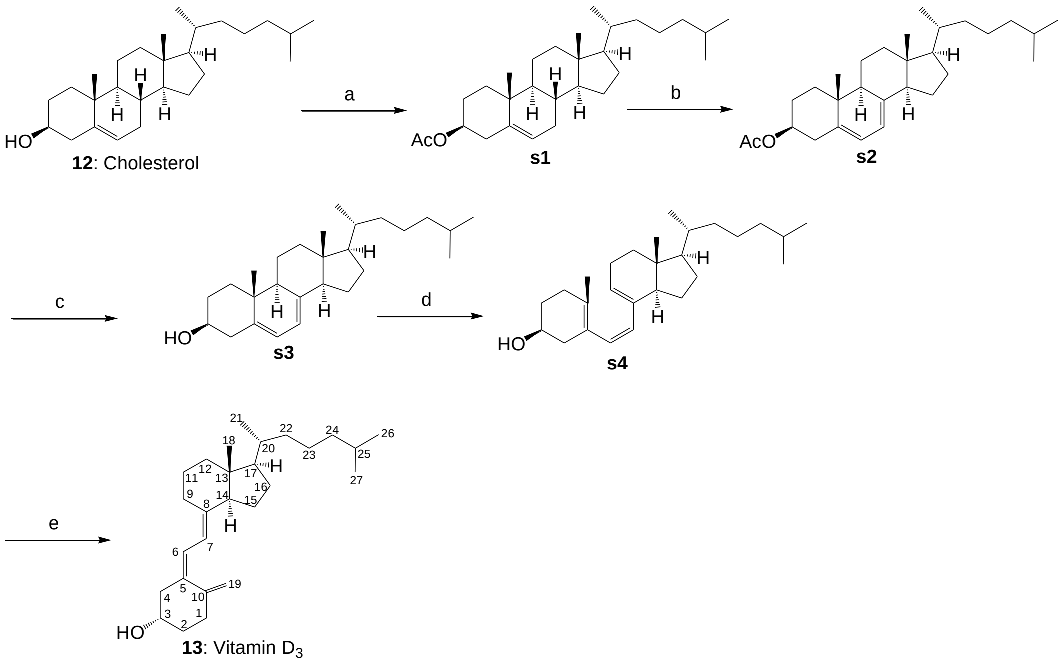

Figure S1. Synthesis of vitamin D_3. (a) Ac_2O, pyridine, 45 °C, 15 h, 98%. (b) 1) *N*-bromosuccinimide (NBS), cyclohexane, reflux, 1 h, 2) 1.0 M Bu_4NF/THF, room temperature, 12 h, 39%. (c) 28% NaOMe in MeOH, CH_2Cl_2/MeOH, room temp, 2 h, 62%. (d) 0.1% 3-*tert*-butyl-4-hydroxyanisole (BHA) in cyclohexane, 280 nm, 9.03 mW/cm^2, room temp, 1 h, 25%. (e) 0.1% BHA in cyclohexane, reflux, 1 h, 41%.

*The experimental details on the synthesis of vitamin D_3 (**13**).*

Cholesterol **12** (3.0 g, 7.76 mmol) was dissolved in pyridine (20 mL), acetic anhydride (5.0 mL, 52.9 mmol) was added, and the mixture was stirred at 45 °C for 15 h. After ice was added, the mixture was stirred for 1 h, extracted with chloroform, washed with 2N aq. HCl, concentrated, and then the product was crystallized with ethanol to obtain compound **s1** (3.25 g, 98%). Compound **s1** (3.0 g, 7.00 mmol) was dissolved in cyclohexane (80 mL) at 65 °C, *N*-bromosuccinimide (NBS; 1.868 g, 10.49 mmol) was added and then stirred under reflux conditions (90 °C) for 1 h. After cooling the reaction to room temperature, water (100 mL) was added and then stirred for 1 h. The mixture was extracted with *n*-hexane, washed with water, concentrated and dried in vacuo. To the resulting mixture, a 1.0 M solution of tetrabutylammonium fluoride in THF (10.5 mL) was added and stirred at room temperature for 12 h. The reaction product was extracted using *n*-hexane, washed with water, concentrated, and then the main product was separated with silica-gel column chromatography (ethyl acetate/*n*-hexane 1:10) to obtain compound **s2** (1.17 g, 39%). Compound **s2** (1.12 g, 2.62 mmol)

was dissolved in dichloromethane (8 mL) and methanol (30 mL), 28% NaOMe in MeOH was added until pH 10 and then stirred for 2 h. After the reaction mixture was concentrated, the main product was separated with silica-gel column chromatography (ethyl acetate/*n*-hexane 1:4) to obtain compound **s3** (0.625 g, 62%). Compound **s3** (20 mg, 0.052 mmol) was dissolved in 0.1% 3-*tert*-butyl-4-hydroxyanisole (BHA) in cyclohexane (2 mL) and transferred to a petri dish. While stirring the mixture in a petri dish covered with a polyvinylidene chloride food wrap, the mixture was irradiated with UV at 280 nm (9.03 mW/cm^2) for 1 h. After the reaction mixture was concentrated, the main product was separated using silica-gel column chromatography (ethyl acetate/*n*-hexane 1:4) to obtain compound **s4** (5.02 mg, 25%). Compound **s4** (5.02 mg, 0.013 mmol) was dissolved in 0.1% BHA in cyclohexane (2 mL) and the mixture was stirred under reflux conditions (100 °C) for 1 h. After the reaction mixture was concentrated, the main product was separated using silica-gel column chromatography (ethyl acetate/*n*-hexane 1:4) to obtain the vitamin D_3 (**13**: 2.08 mg, 41%). Vitamin D_3 (**13**); ^{1}H-NMR (400 MHz, $CDCl_3$), δ = 0.54 (s, 3H, Me-18), 0.86 (d, 3H, J = 6.6 Hz, Me-26 or Me-27), 0.87 (d, 3H, J = 6.6 Hz, Me-26 or Me-27), 0.92 (d, 3H, J = 6.4 Hz, Me-21), 1.01 (m, 1H, H-22a), 1.08 – 1.18 (m, 3H, H-23a, H-24a, H-24b), 1.23 – 1.41 (m, 6H, H-12a, H-16a, H-17, H-20, H-22b, H-23b), 1.43 – 1.58 (m, 4H, H-11a, H-15a, H-15b, H-25), 1.63 – 1.73 (m, 3H, H-2a, H-9a, H-11b), 1.81 – 2.03 (m, 4H, H-2b, H-12b, H-14, H-16b), 2.18 (m, 1H, H-1a), 2.28 (dd, 1H, J = 12.9, 7.3 Hz, H-4a), 2.40 (m, 1H, H-1b), 2.57 (dd, 1H, J = 12.9, 3.2 Hz, H-4b), 2.82 (m, 1H, H-9b), 3.95 (m, 1H, H-3), 4.82 (broad d, 1H, H-19a), 5.05 (m, 1H, H-19b), 6.03 (d, 1H, J = 11.2 Hz, H-7), 6.23 (d, 1H, J = 11.2 Hz, H-6); ^{13}C-NMR (100 MHz, $CDCl_3$), δ = 12.0 (C-18), 18.8 (C-21), 22.3 (C-15), 22.5 (C-26 or C-27), 22.8 (C-26 or C-27), 23.6 (C-11), 23.9 (C-23), 27.6 (C-16), 28.0 (C-25), 29.0 (C-9), 31.9 (C-1), 35.2 (C-2), 36.1 (C-20 and C-22), 39.5 (C-24), 40.5 (C-12), 45.86 (C-4 or C-13), 45.93 (C-4 or C-13), 56.4 (C-14), 56.6 (C-17), 69.2 (C-3), 112.4 (C-19), 117.5 (C-7), 122.5 (C-6), 135.0 (C-5), 142.4 (C-8), 145.1 (C-10).

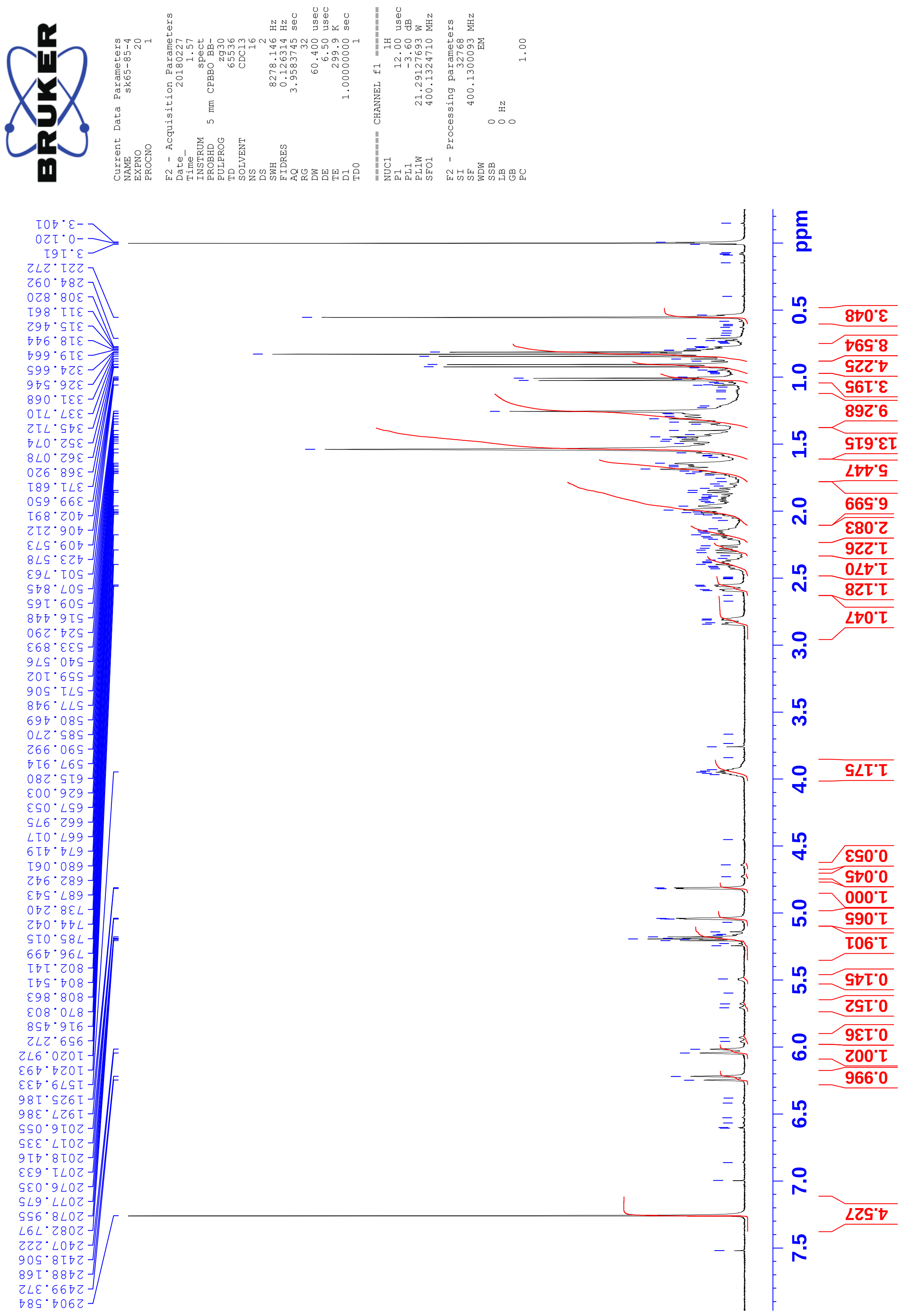

Figure S2. ^{1}H NMR spectrum of vitamin D_2 (**9**).

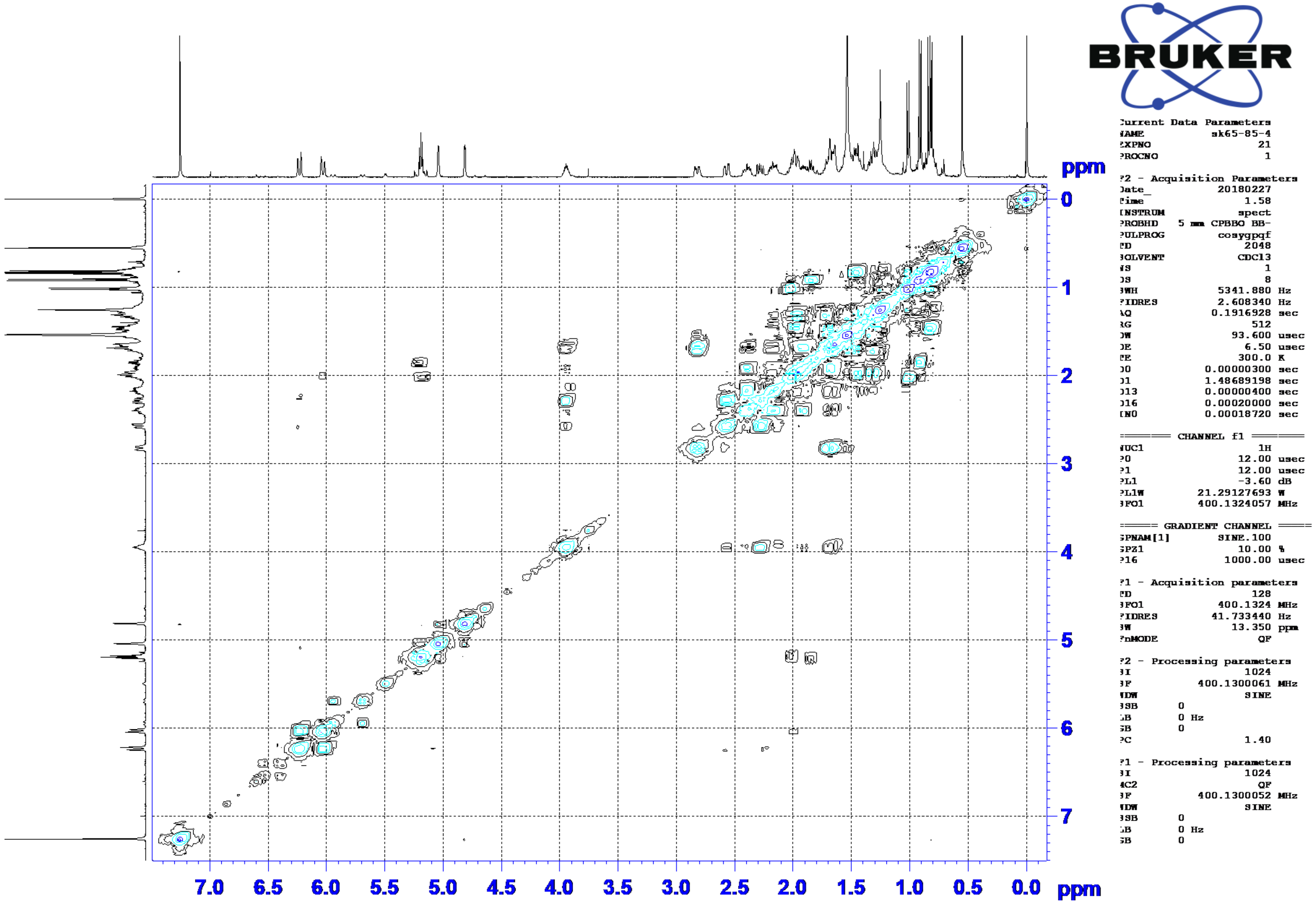

Figure S3. $^{1}H^{1}H$ COSY NMR spectrum of vitamin D_2 (**9**).

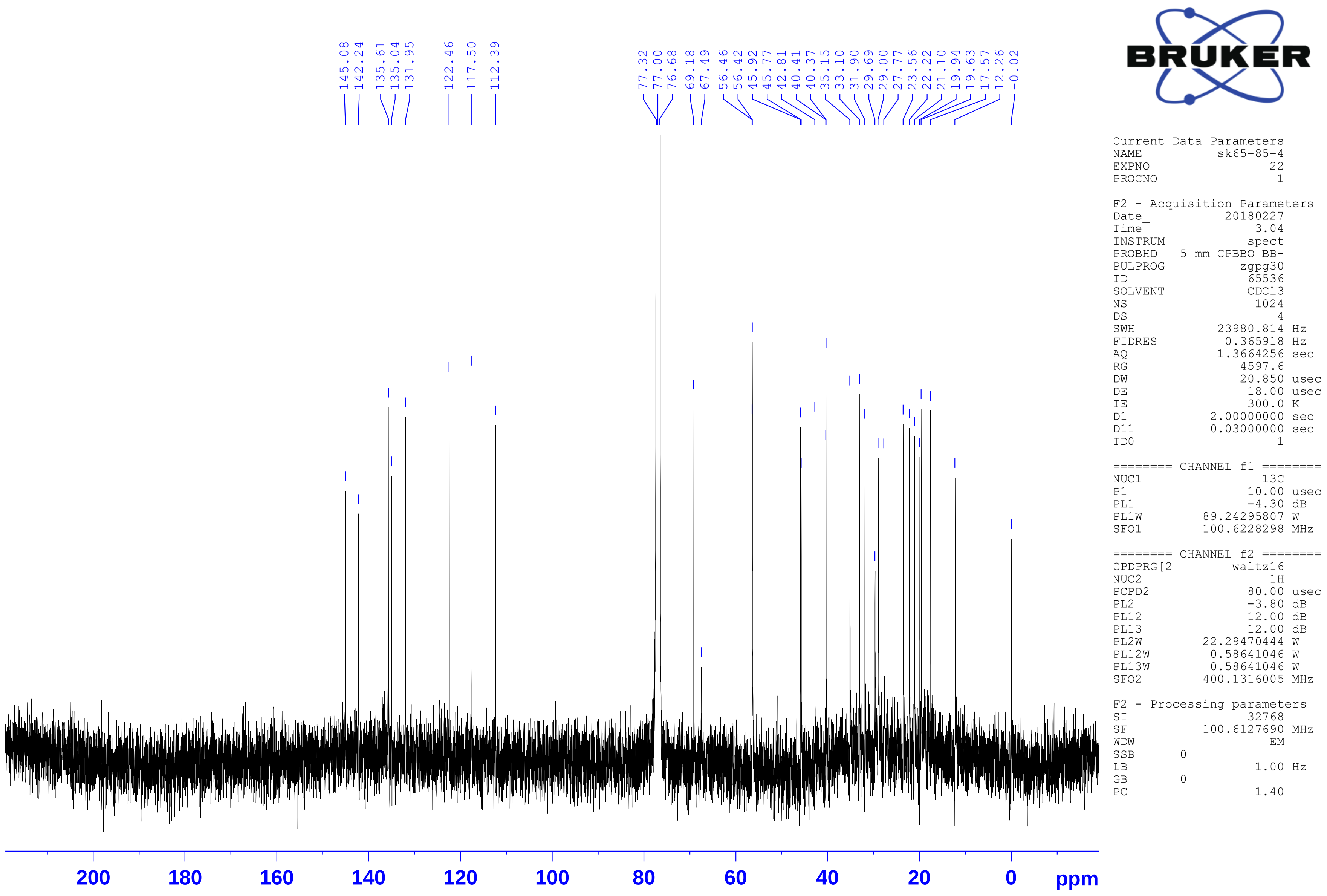

Figure S4. ^{13}C NMR spectrum of vitamin D_2 (**9**).

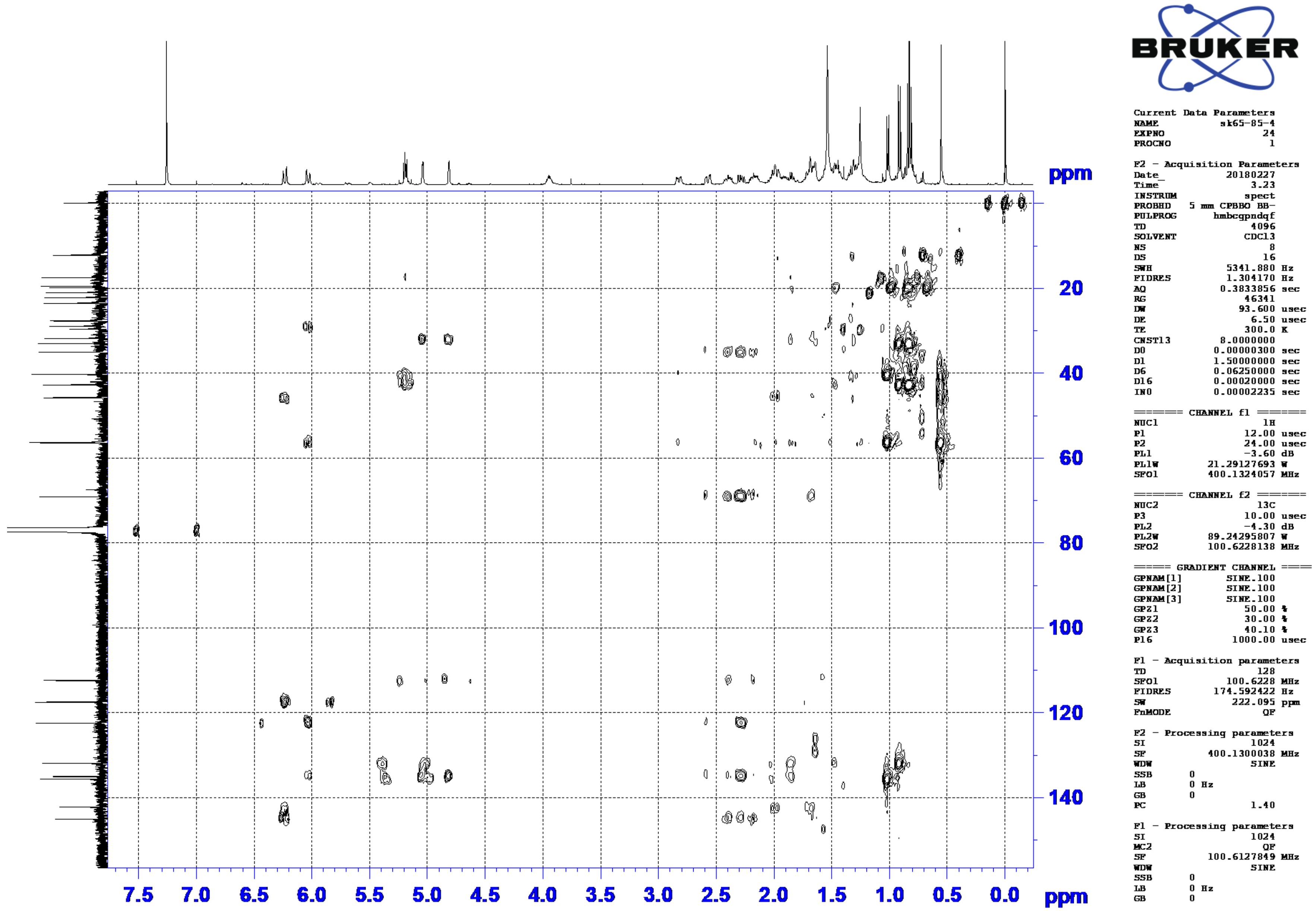

Figure S5. HMBC NMR spectrum of vitamin D_2 (**9**).

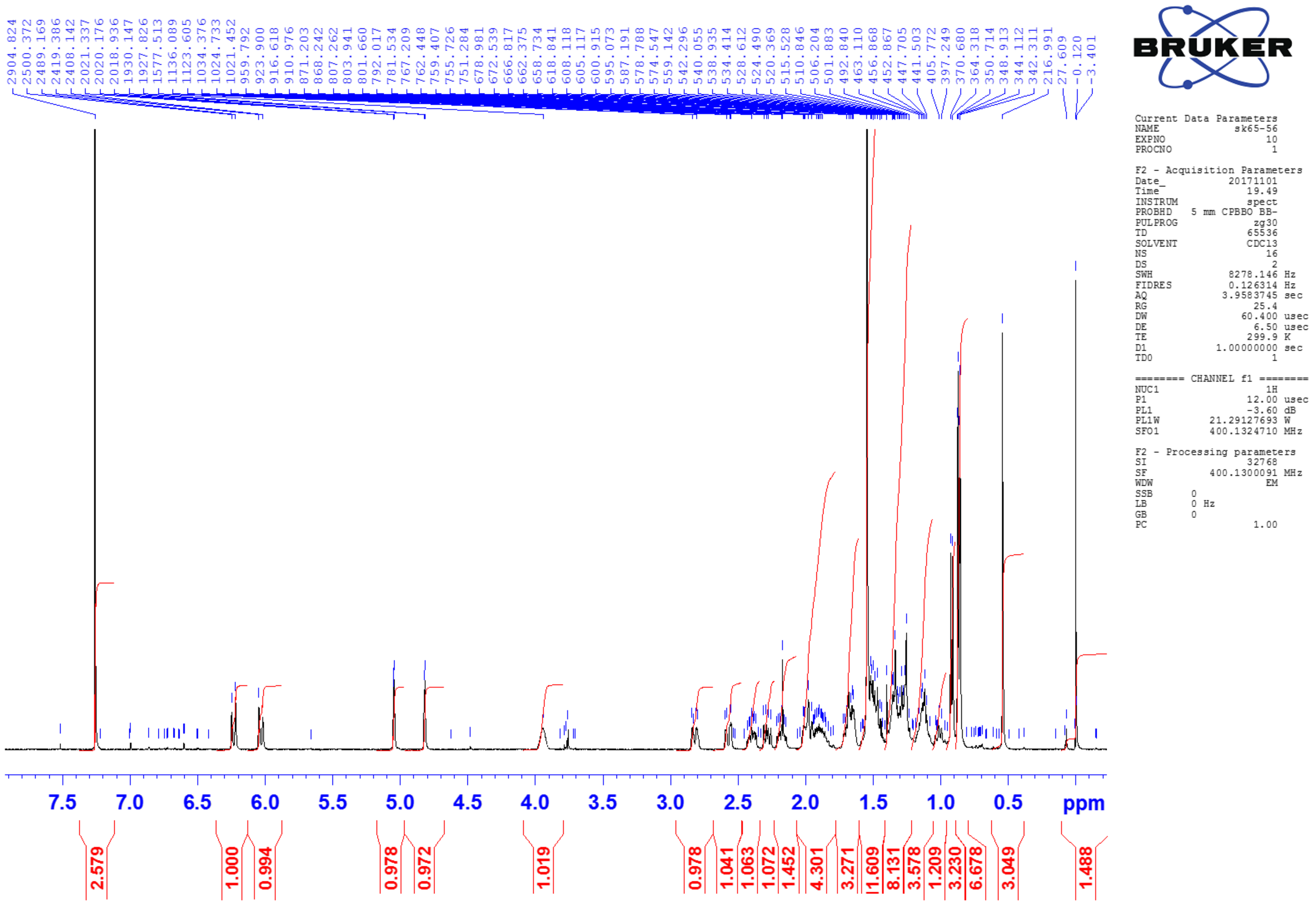

Figure S6. ^{1}H NMR spectrum of vitamin D_3 (**13**).

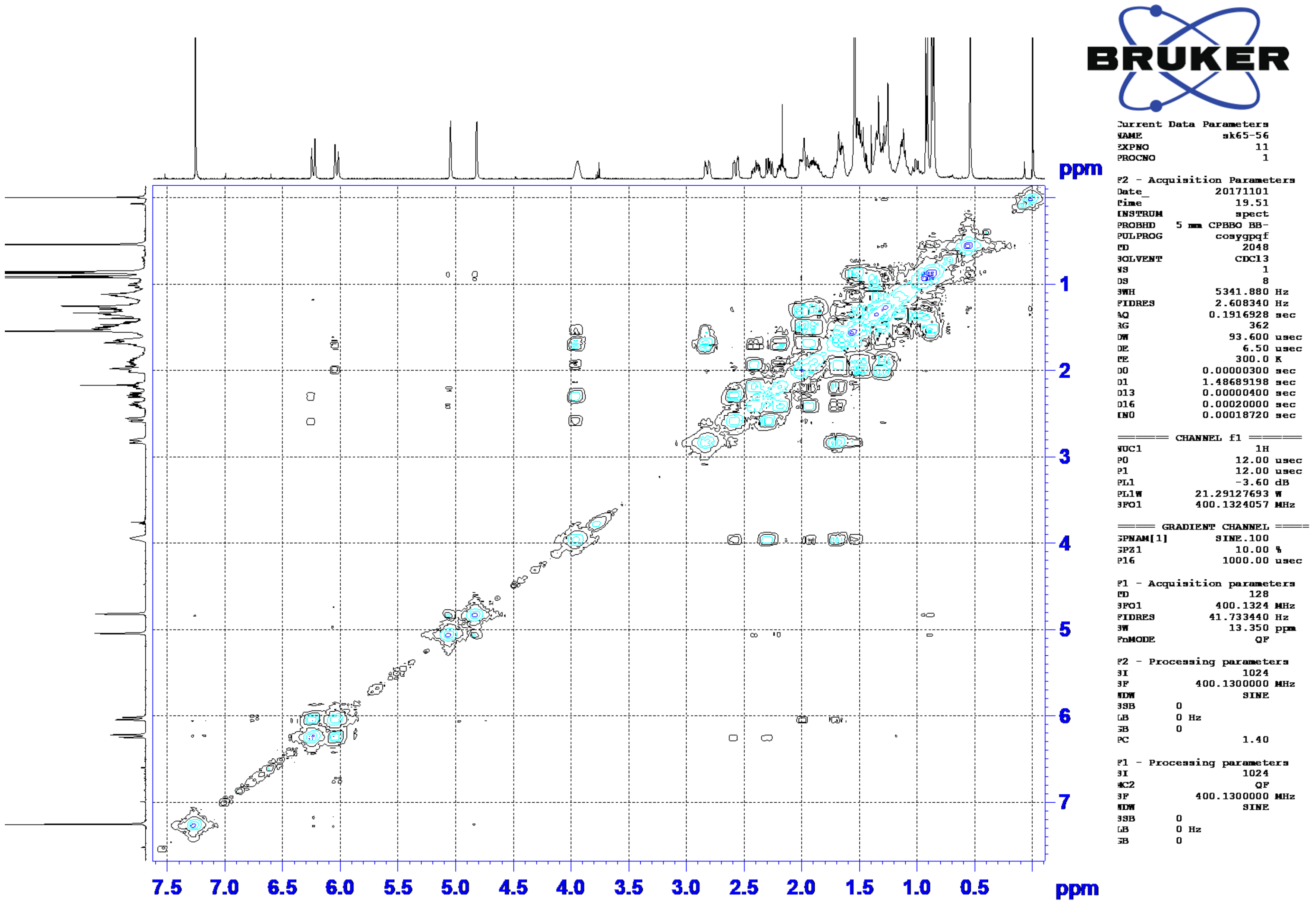

Figure S7. $^1H^1H$ COSY NMR spectrum of vitamin D_3 (**13**).

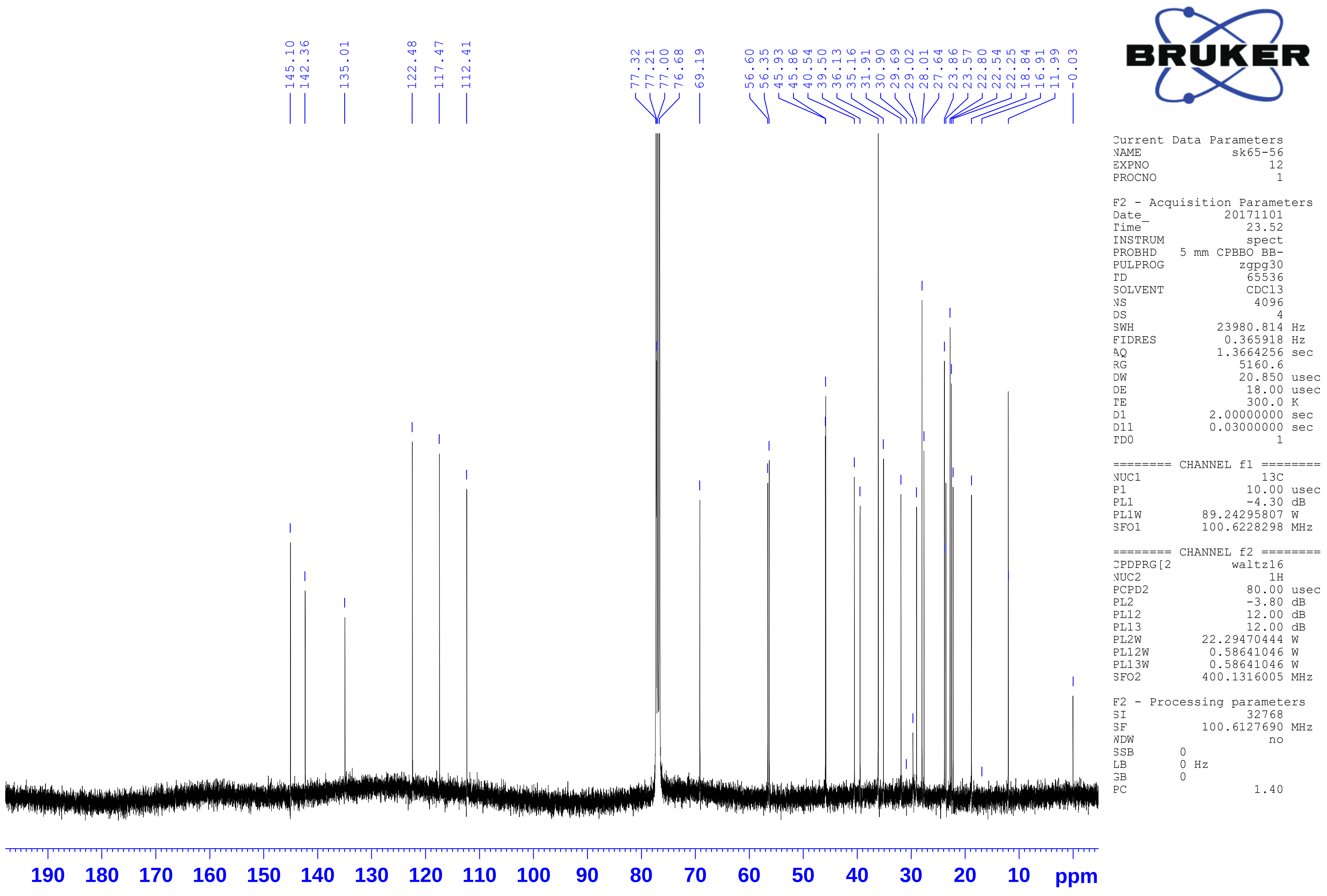

Figure S8. ^{13}C NMR spectrum of vitamin D_3 (**13**).

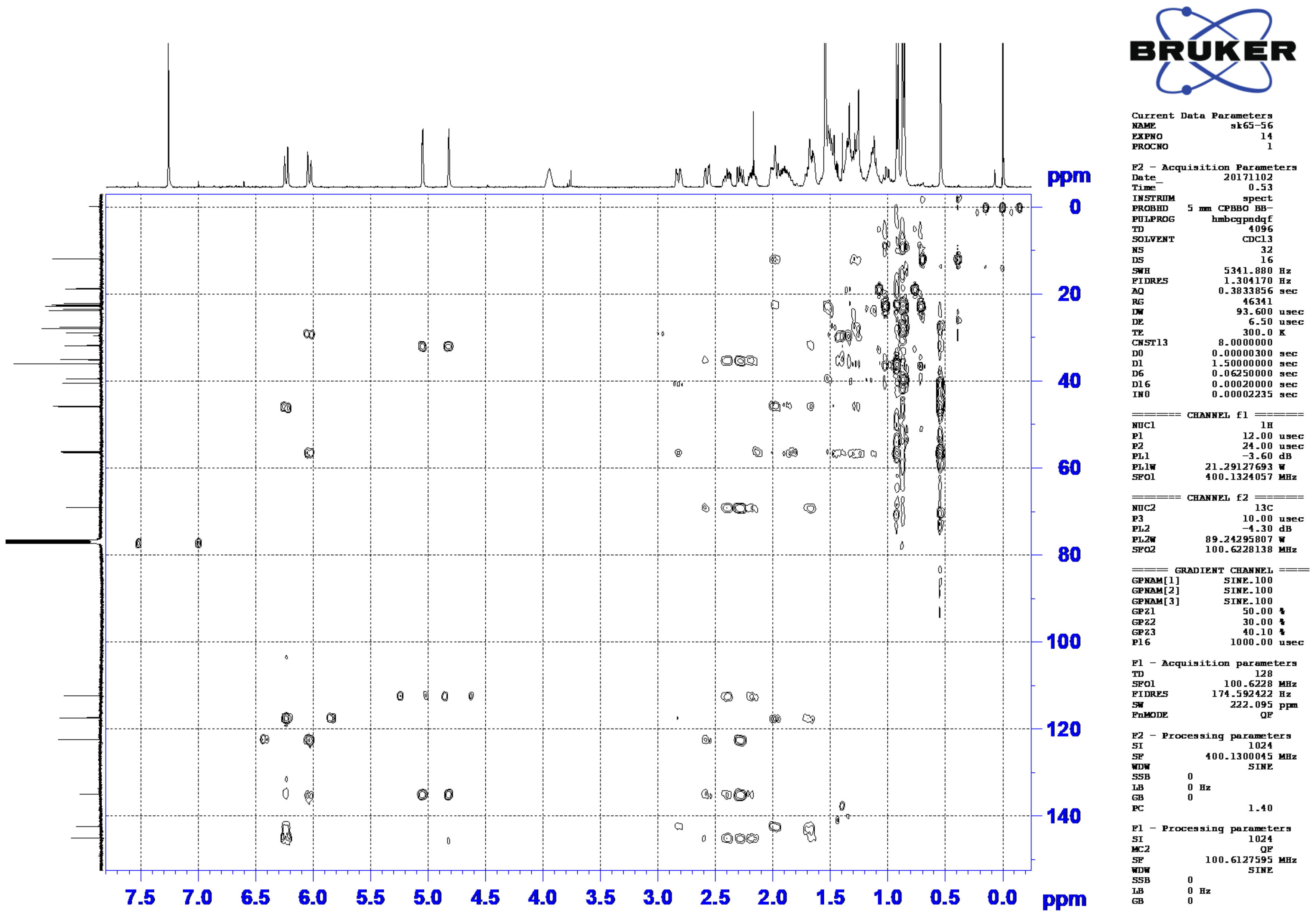

Figure S9. HMBC NMR spectrum of vitamin D_3 (**13**).

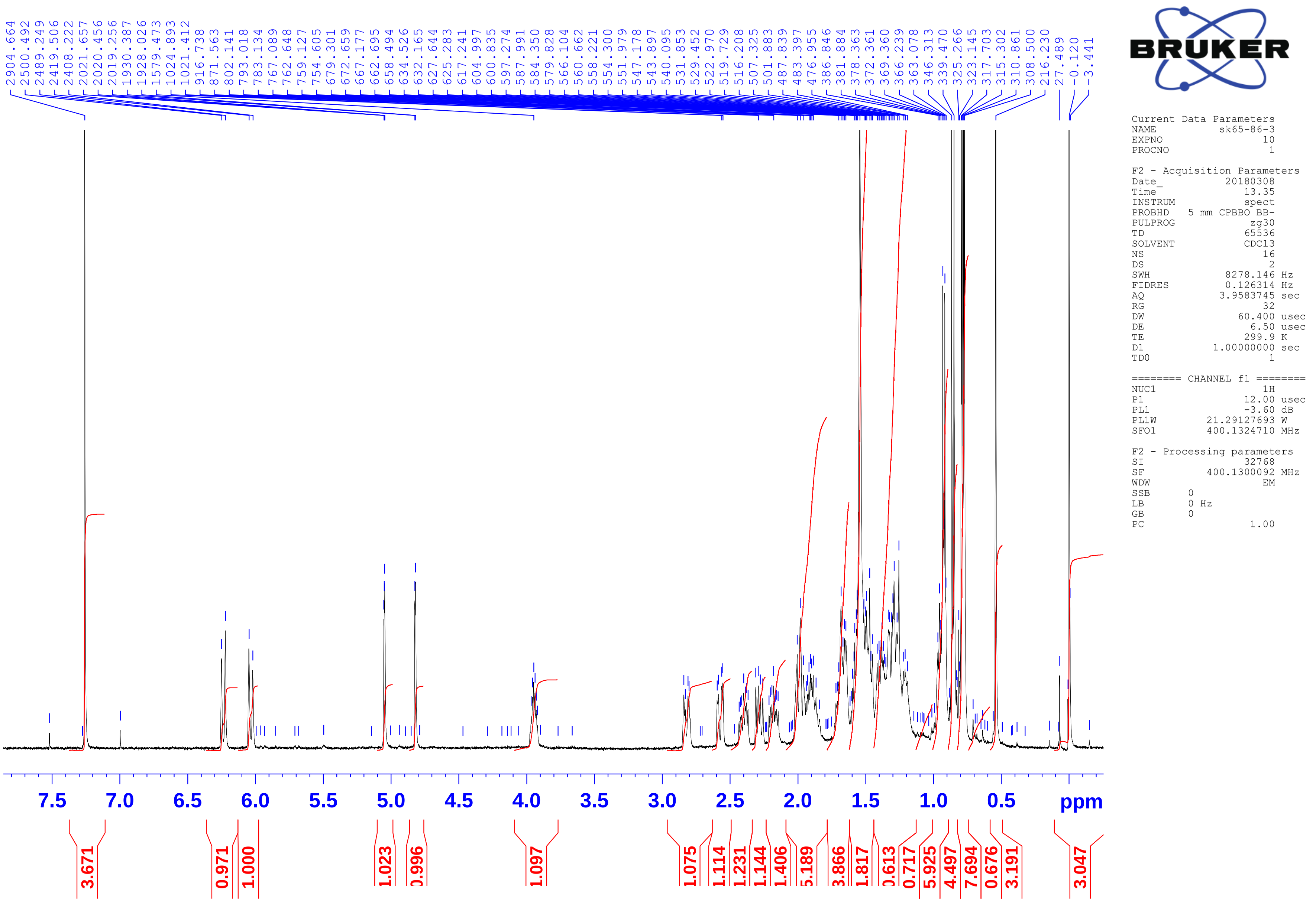

Figure S10. ^{1}H NMR spectrum of vitamin D_4 (**10**).

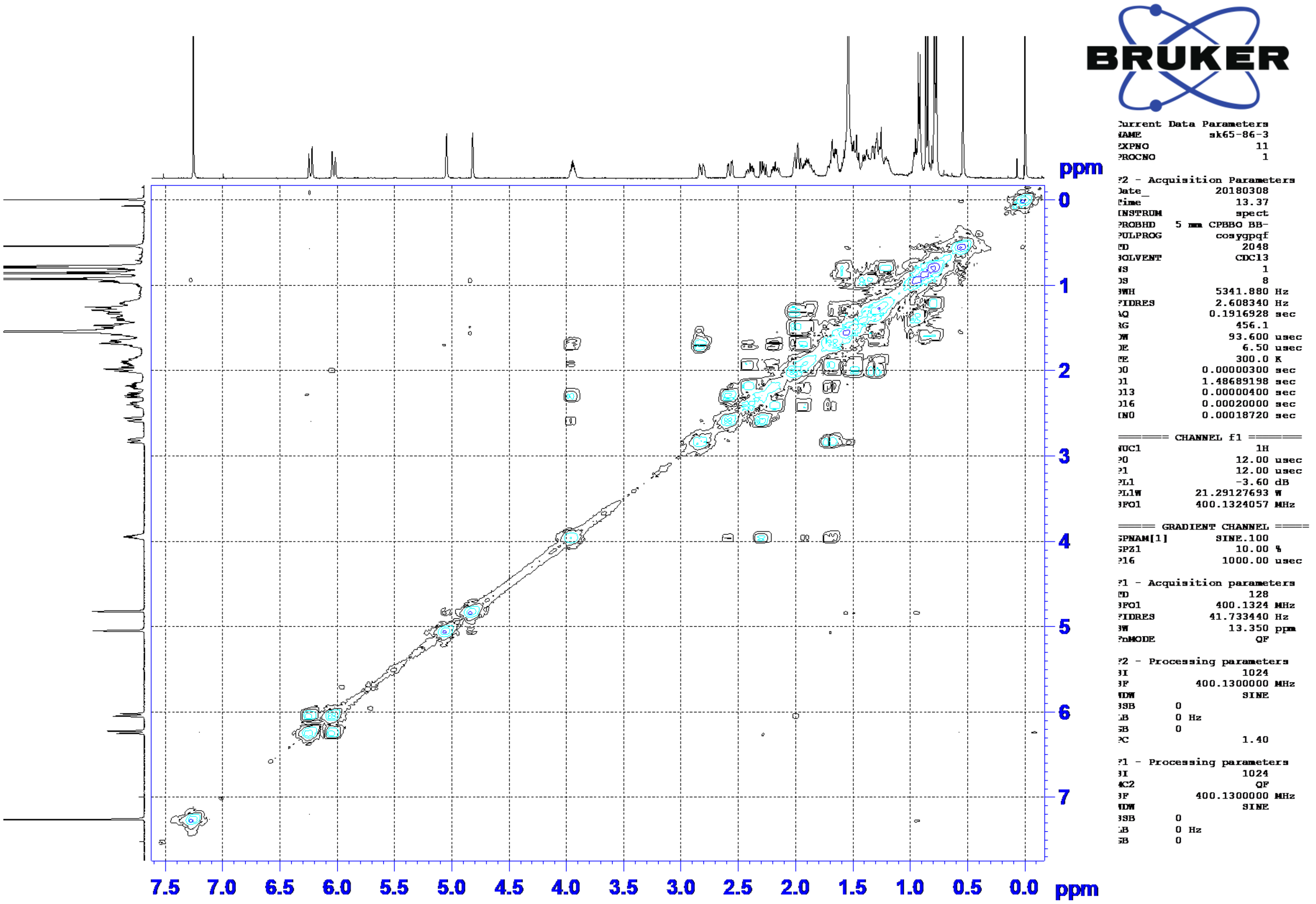

Figure S11. $^{1}H^{1}H$ COSY NMR spectrum of vitamin D_4 (**10**).

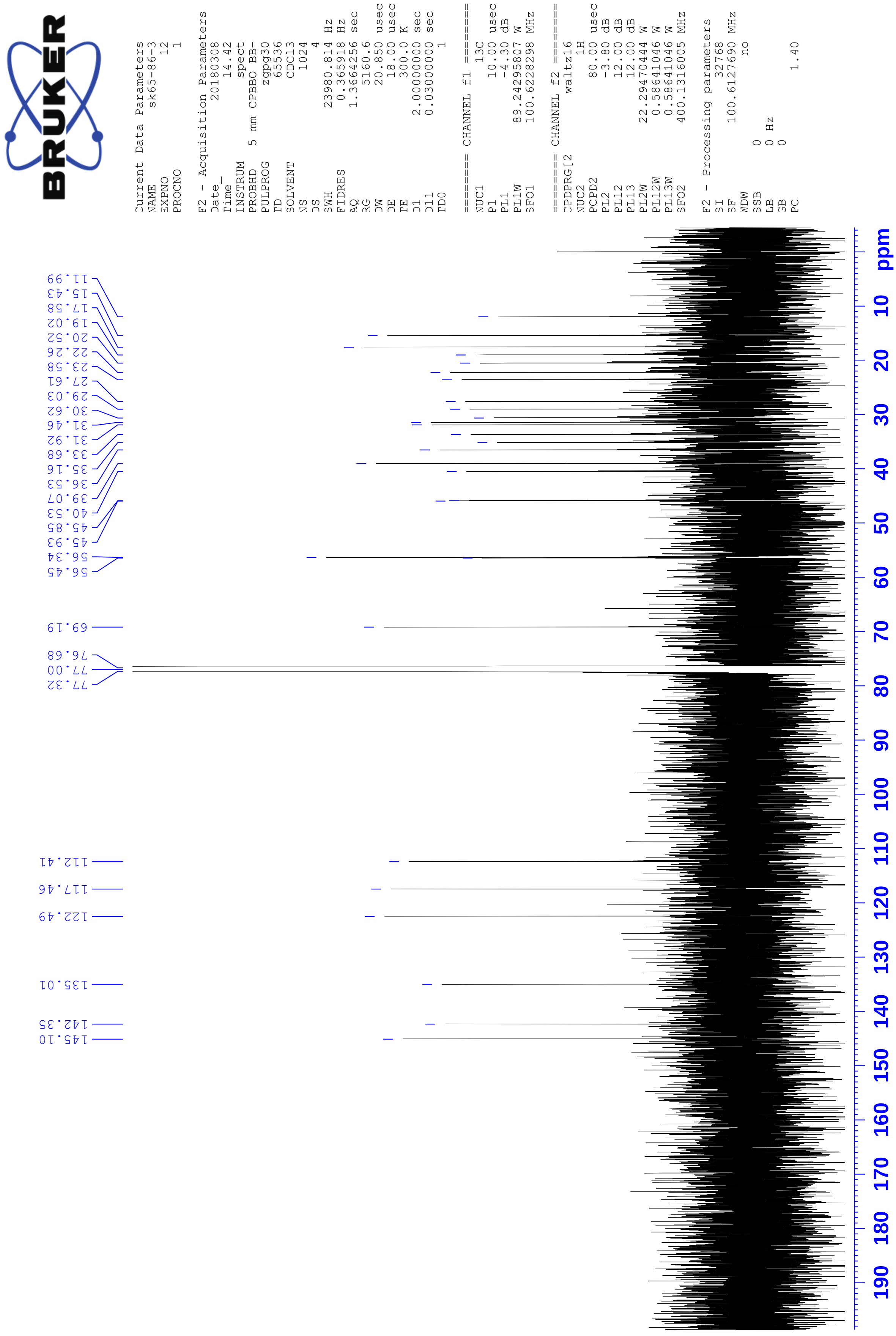

Figure S12. ^{13}C NMR spectrum of vitamin D_4 (10).

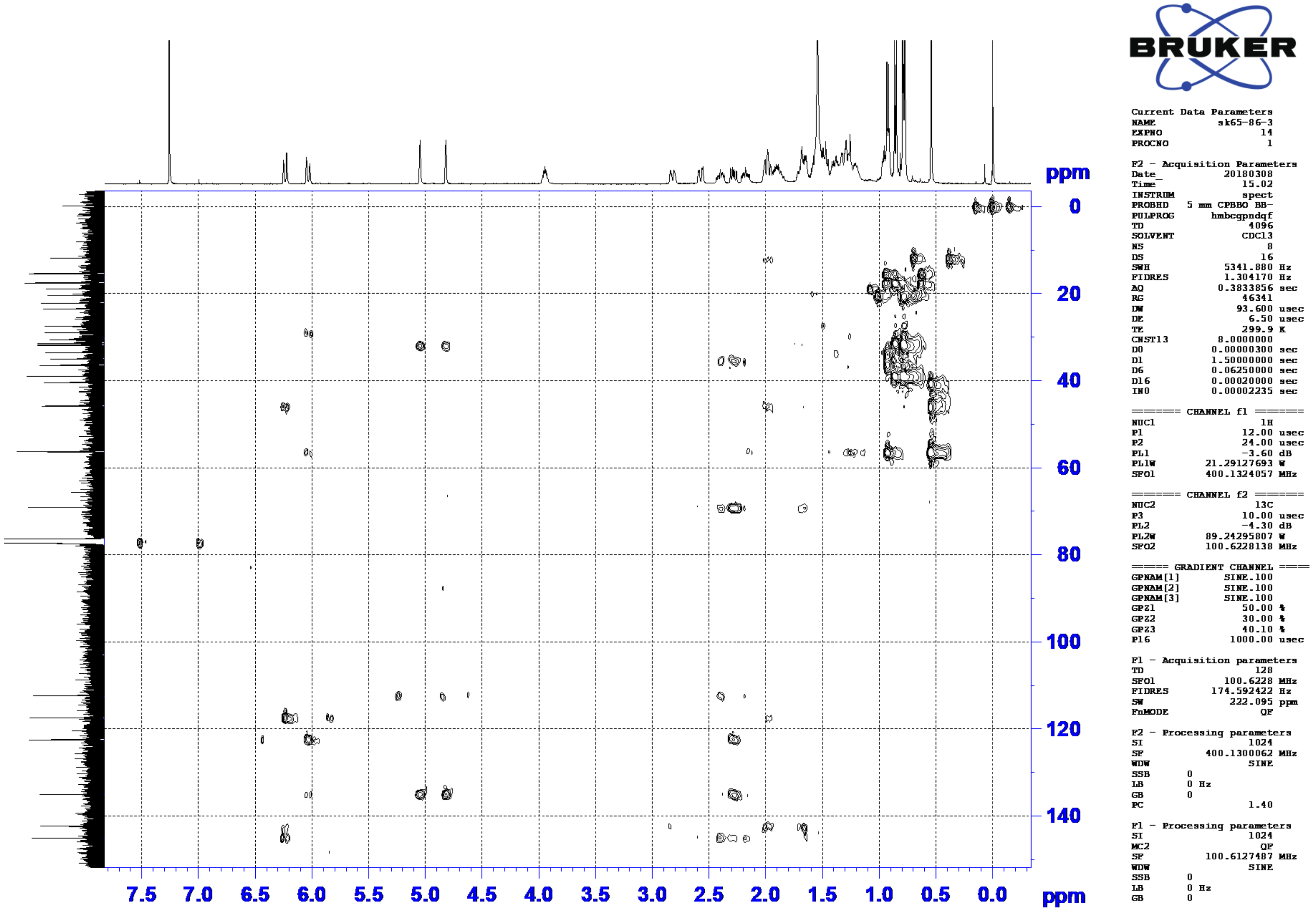

Figure S13. HMBC NMR spectrum of vitamin D_4 (**10**).

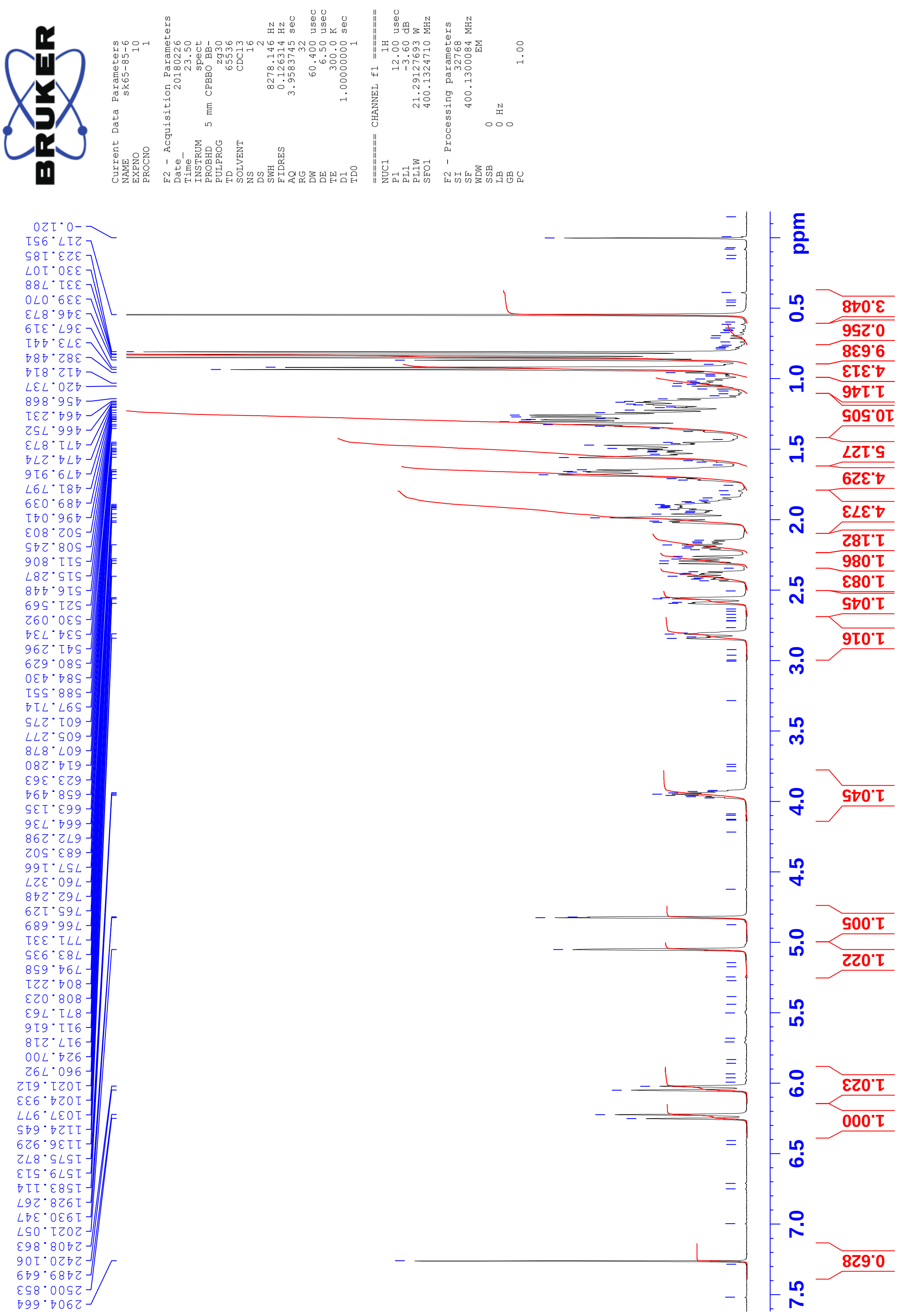

Figure S14. ^{1}H NMR spectrum of vitamin D_5 (**6**).

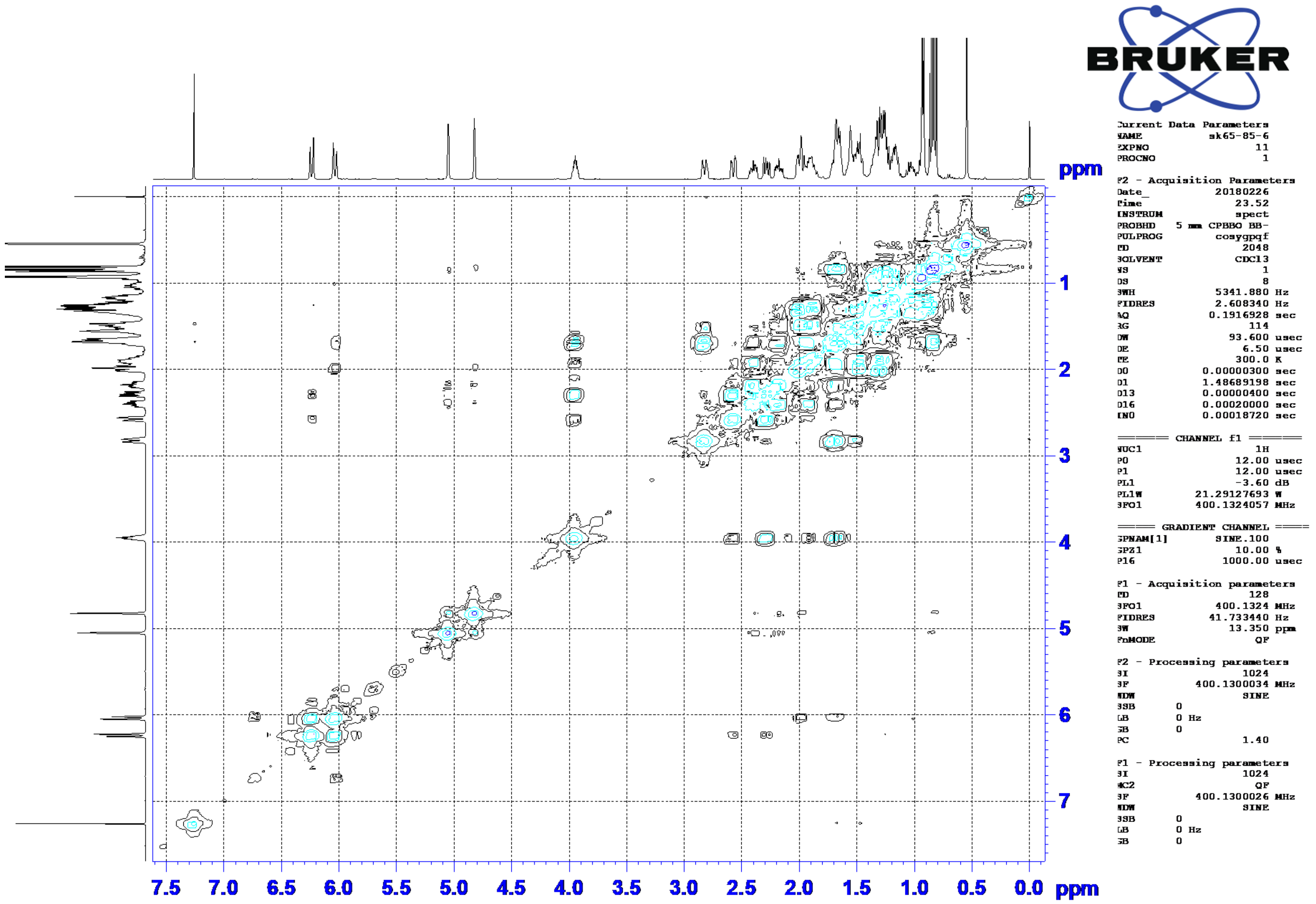

Figure S15. $^{1}H^{1}H$ COSY NMR spectrum of vitamin D_5 (**6**).

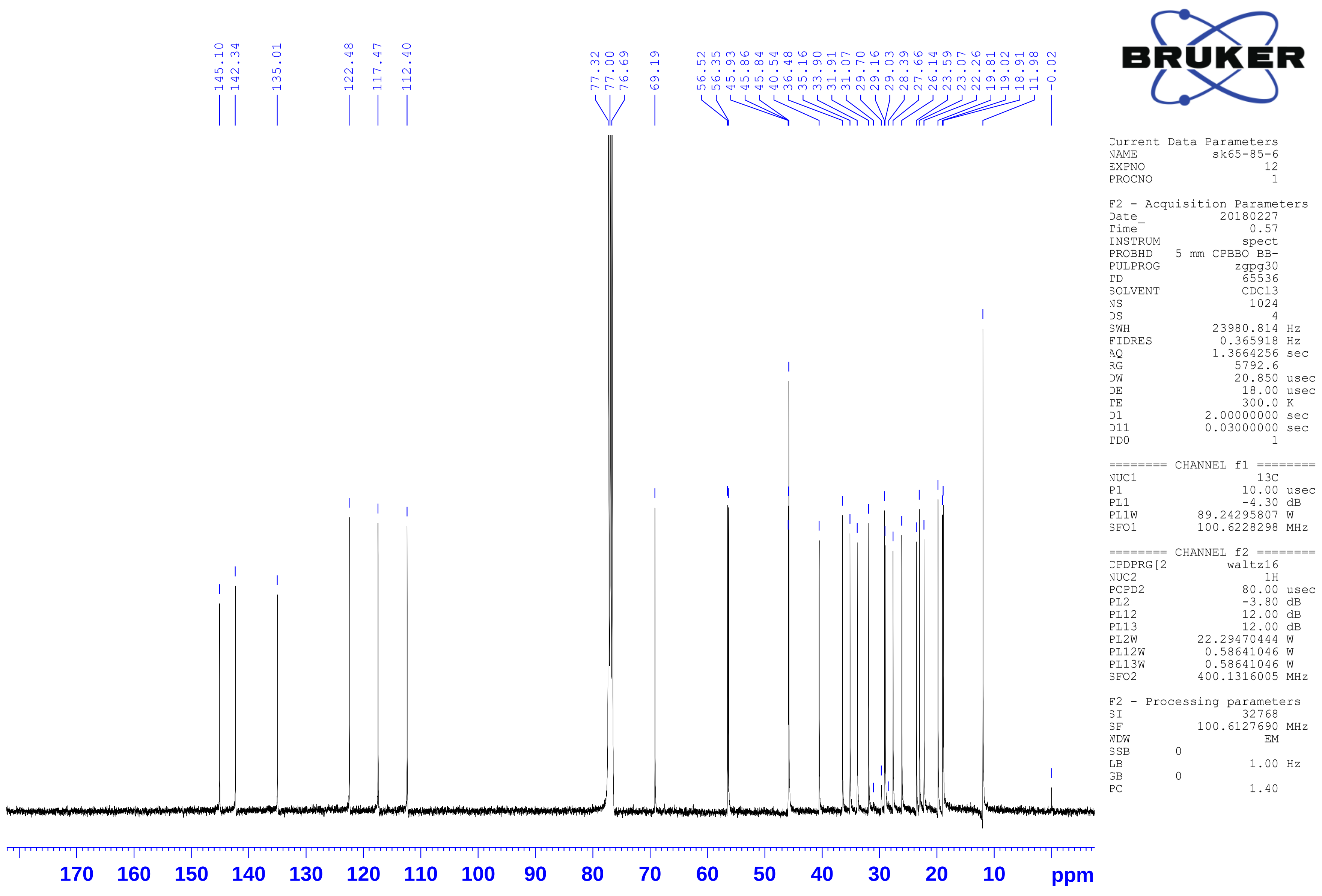

Figure S16. ^{13}C NMR spectrum of vitamin D_5 (**6**).

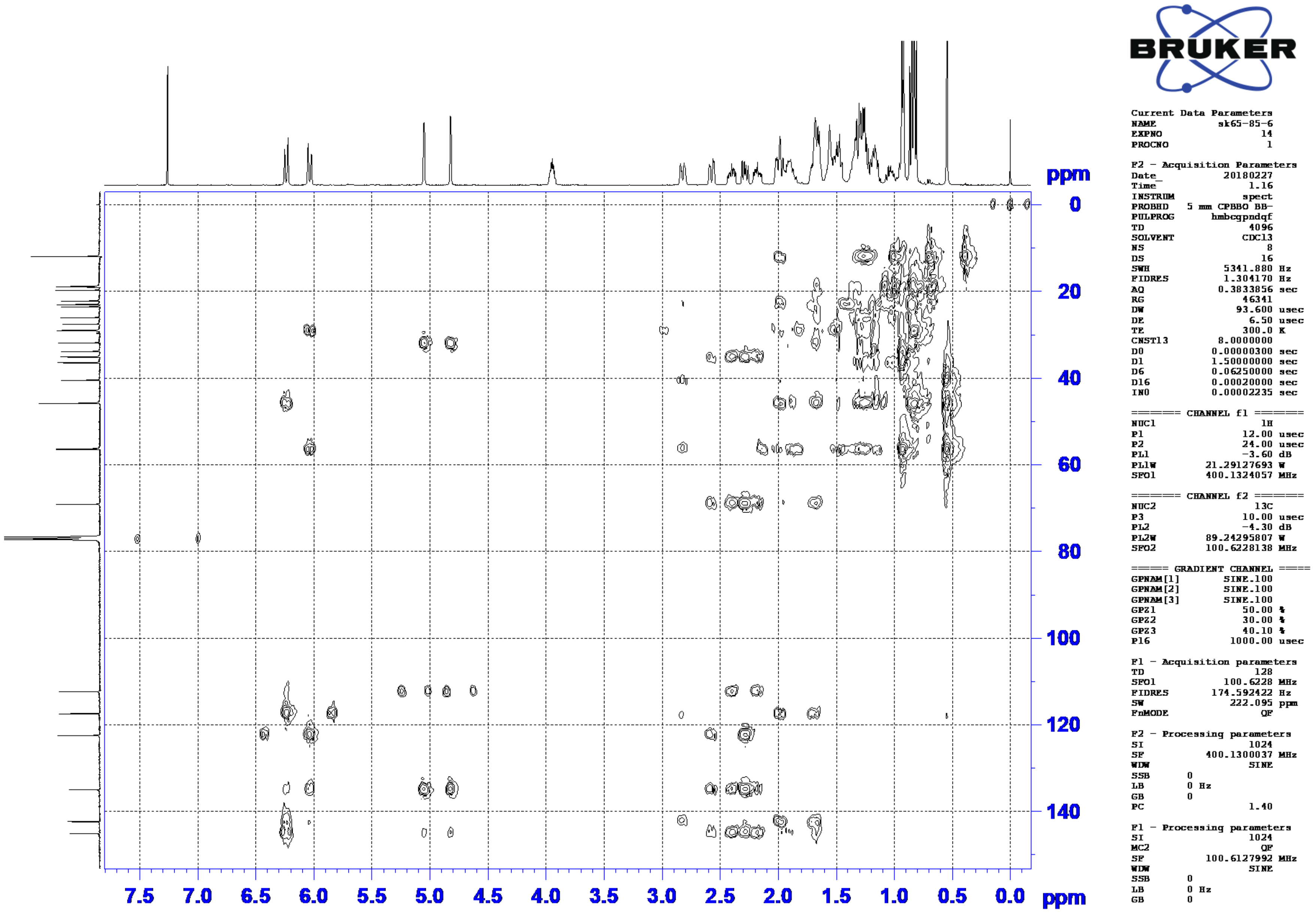

Figure S17. HMBC NMR spectrum of vitamin D_5 (**6**).

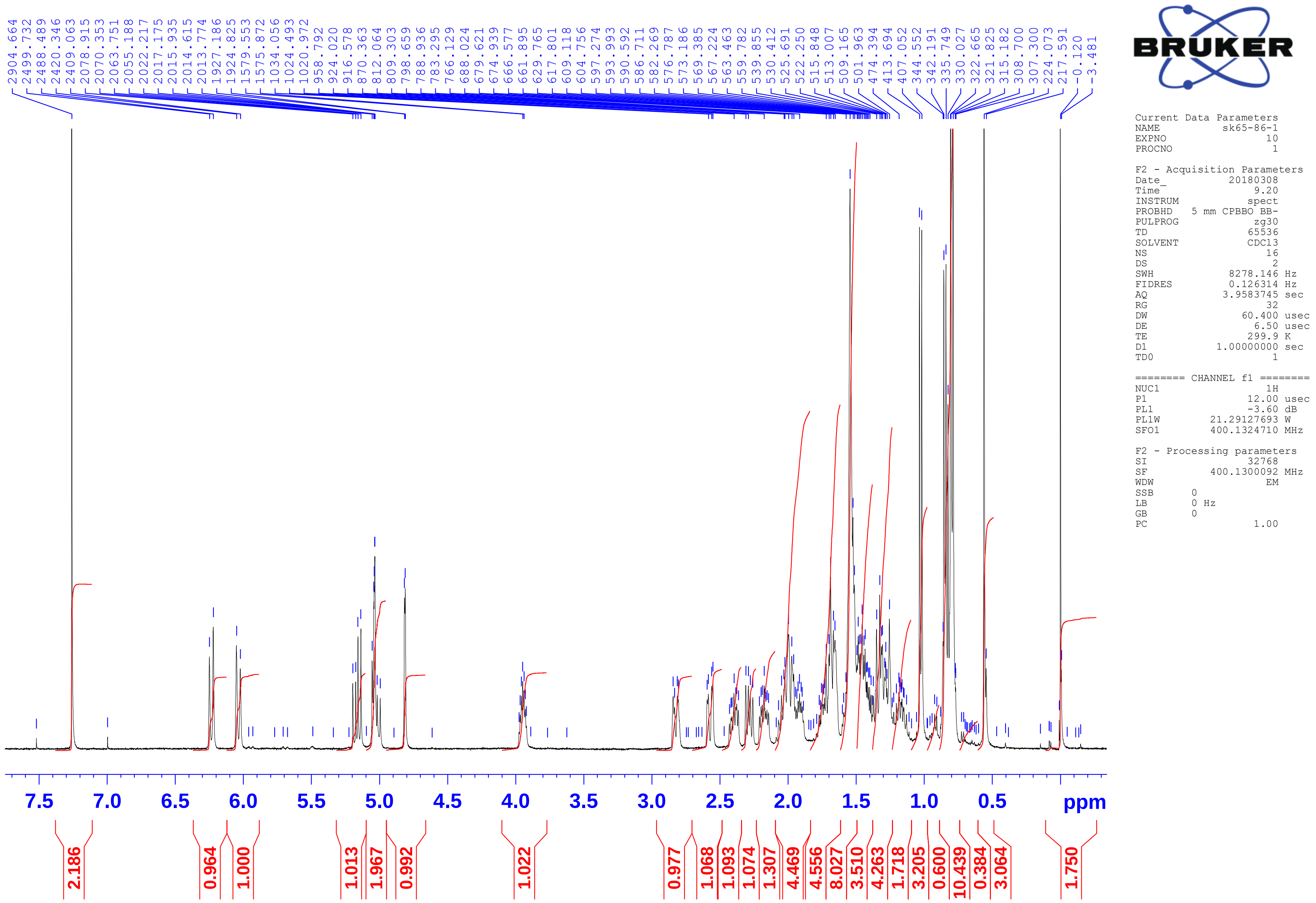

Figure S18. ^{1}H NMR spectrum of vitamin D_6 (**8**).

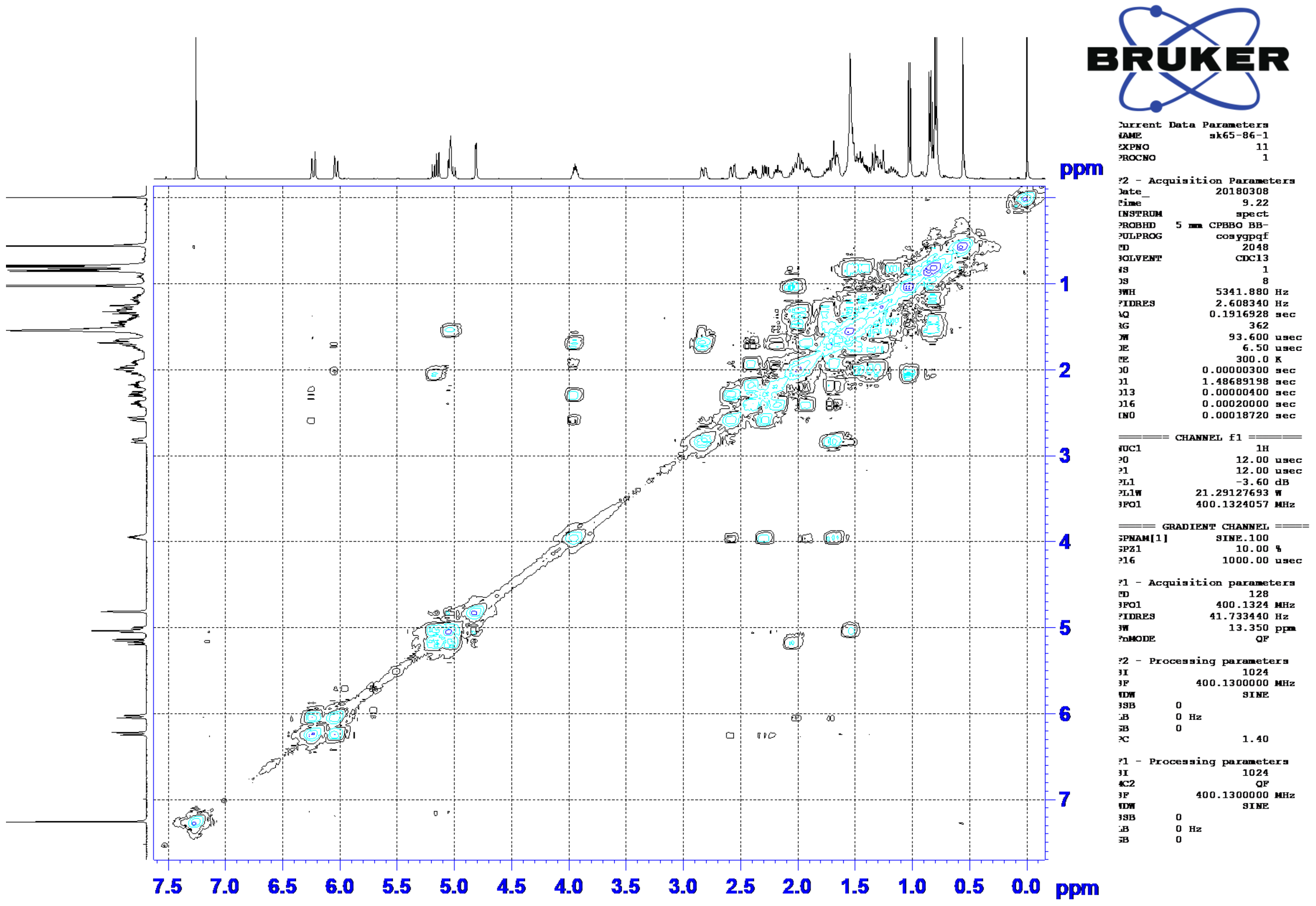

Figure S19. $^1H^1H$ COSY NMR spectrum of vitamin D_6 (**8**).

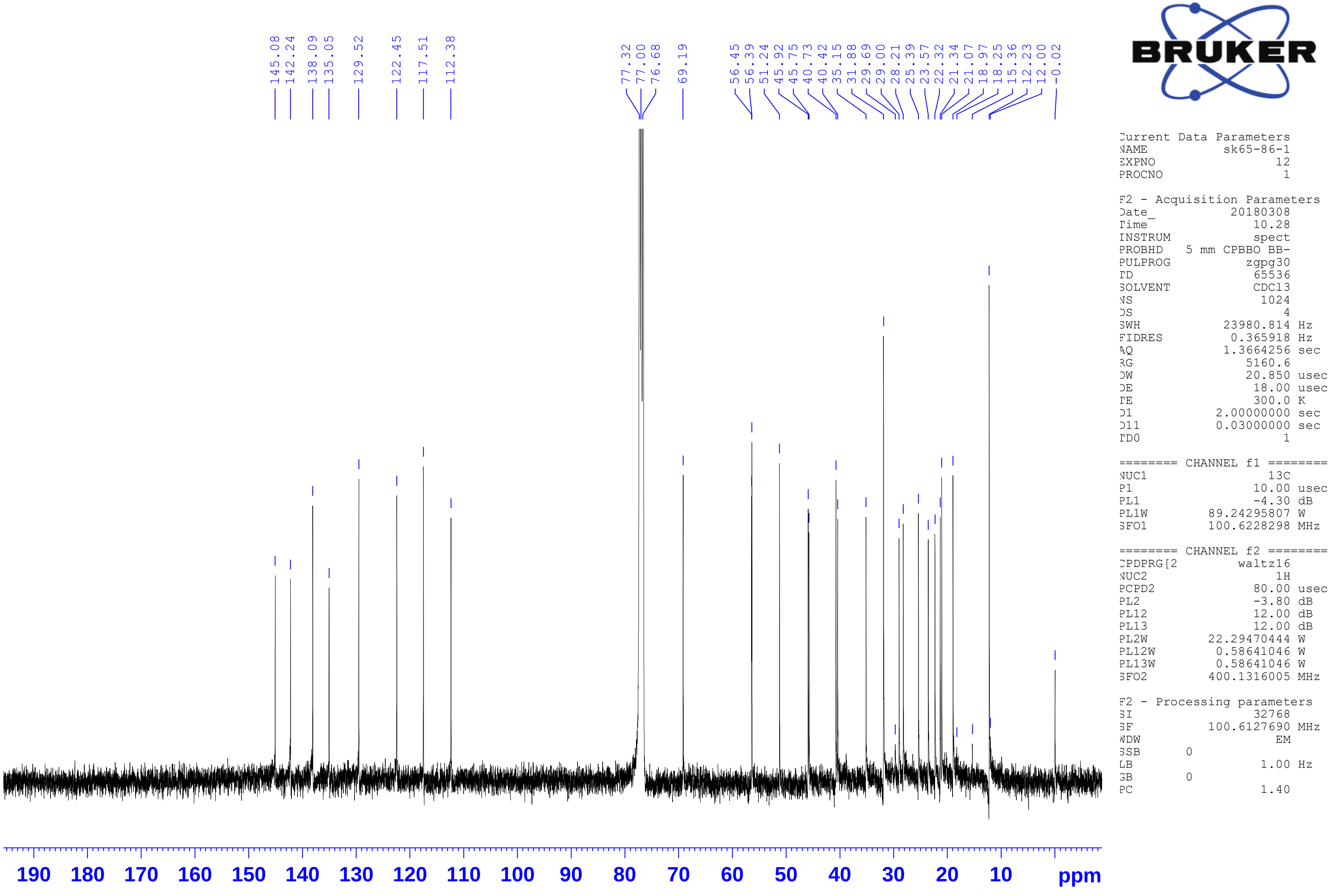

Figure S20. ^{13}C NMR spectrum of vitamin D_6 (**8**).

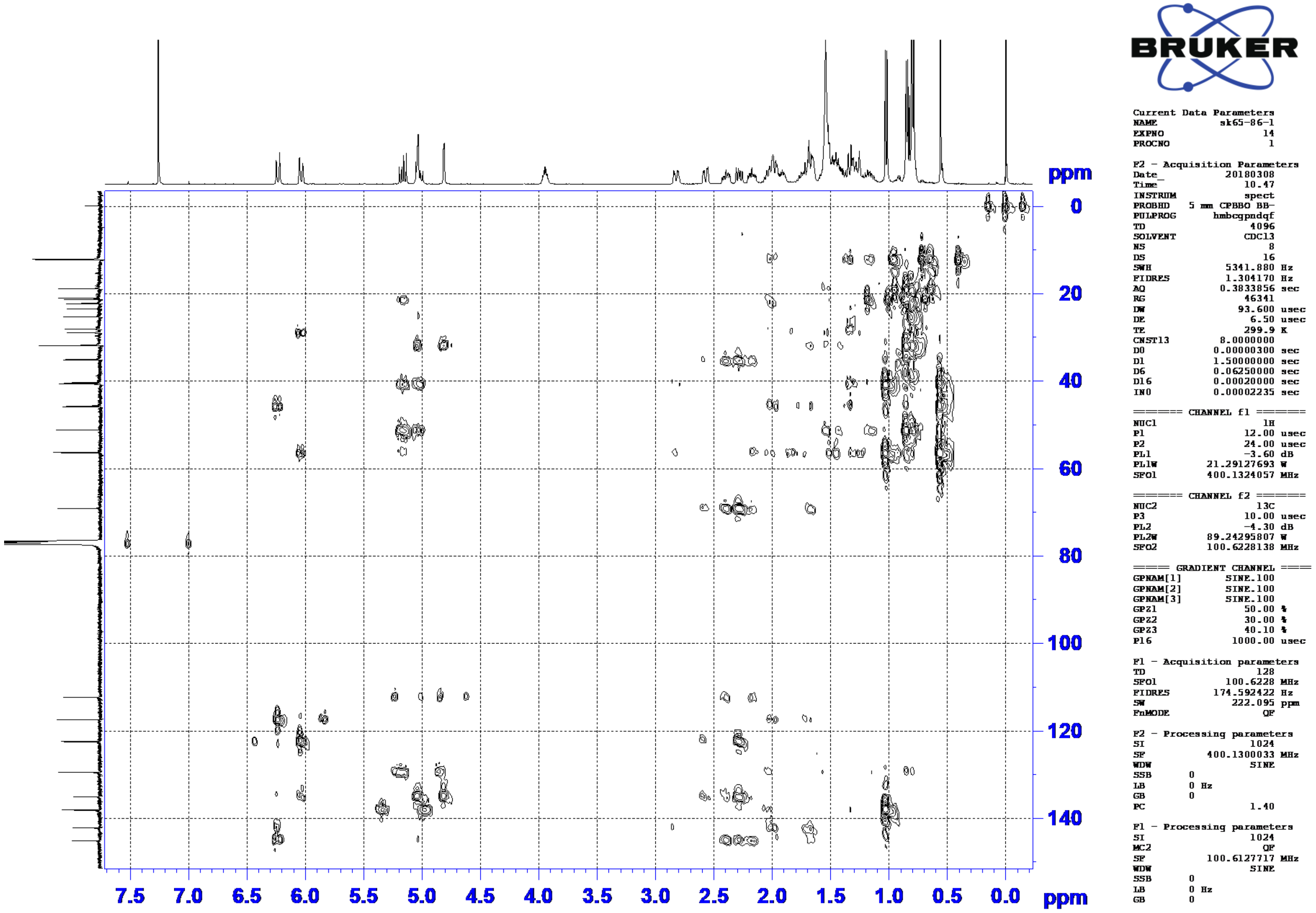

Figure S21. HMBC NMR spectrum of vitamin D_6 (8).

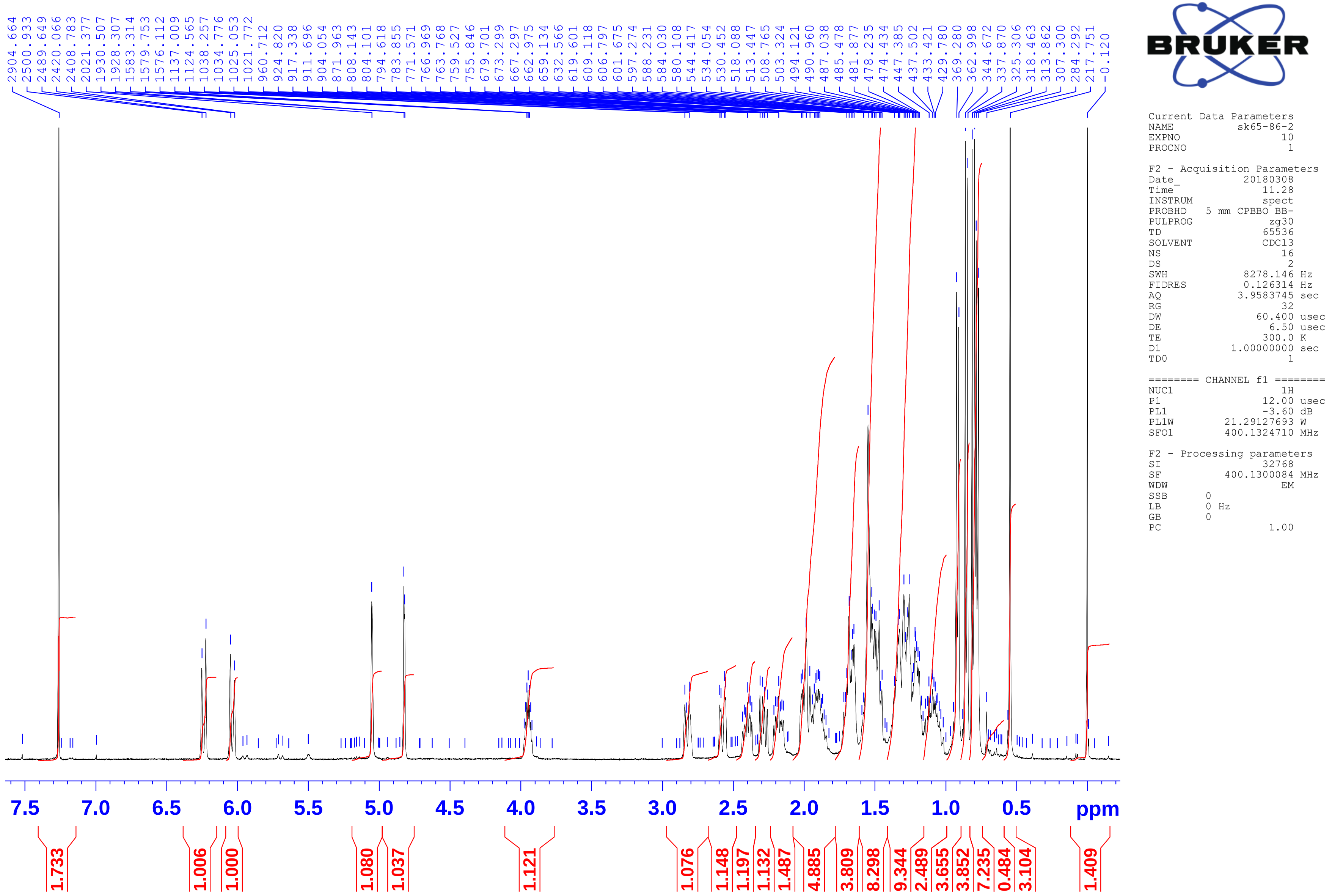

Figure S22. ^{1}H NMR spectrum of vitamin D_7 (7).

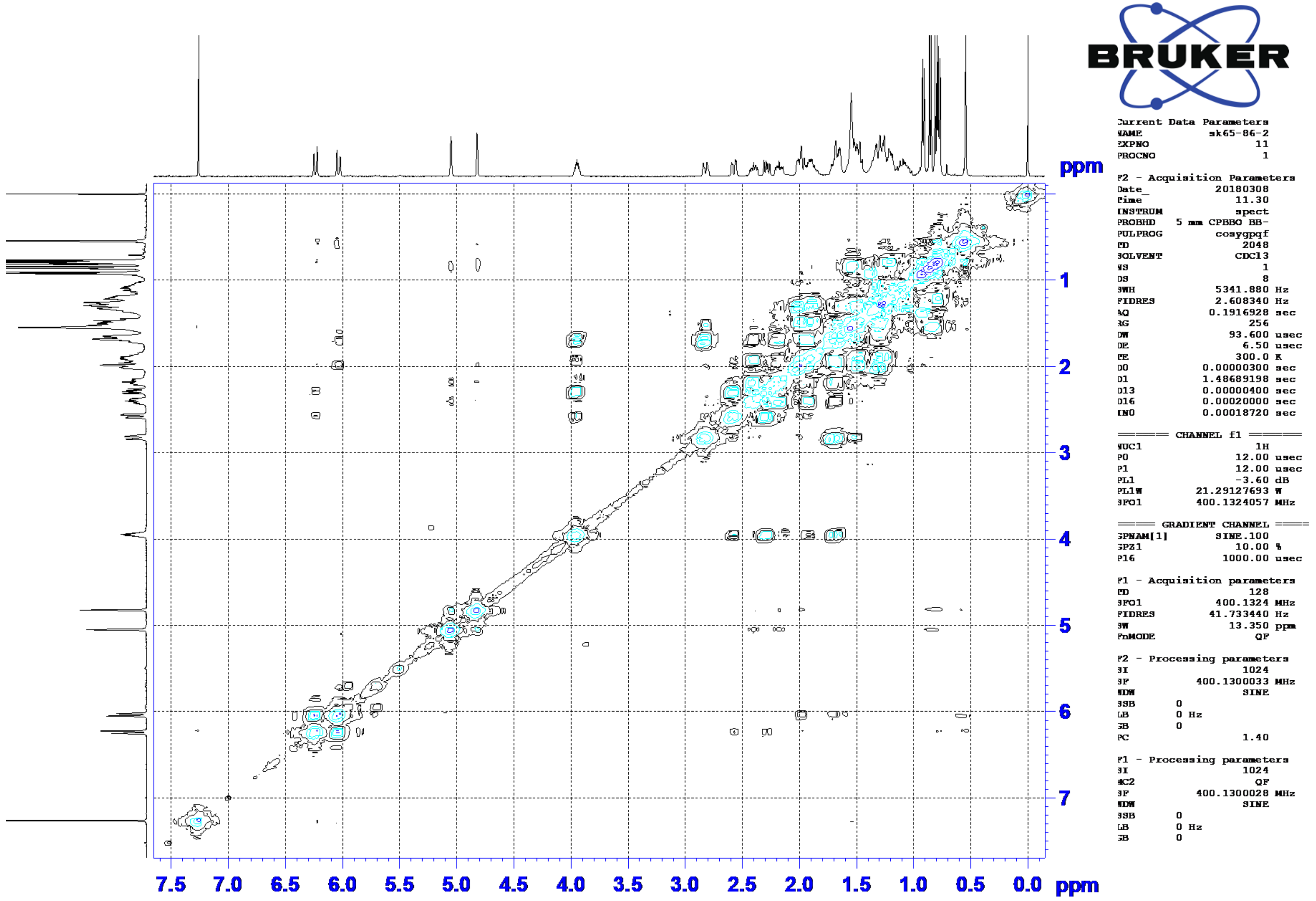

Figure S23. $^{1}H^{1}H$ COSY NMR spectrum of vitamin D_7 (**7**).

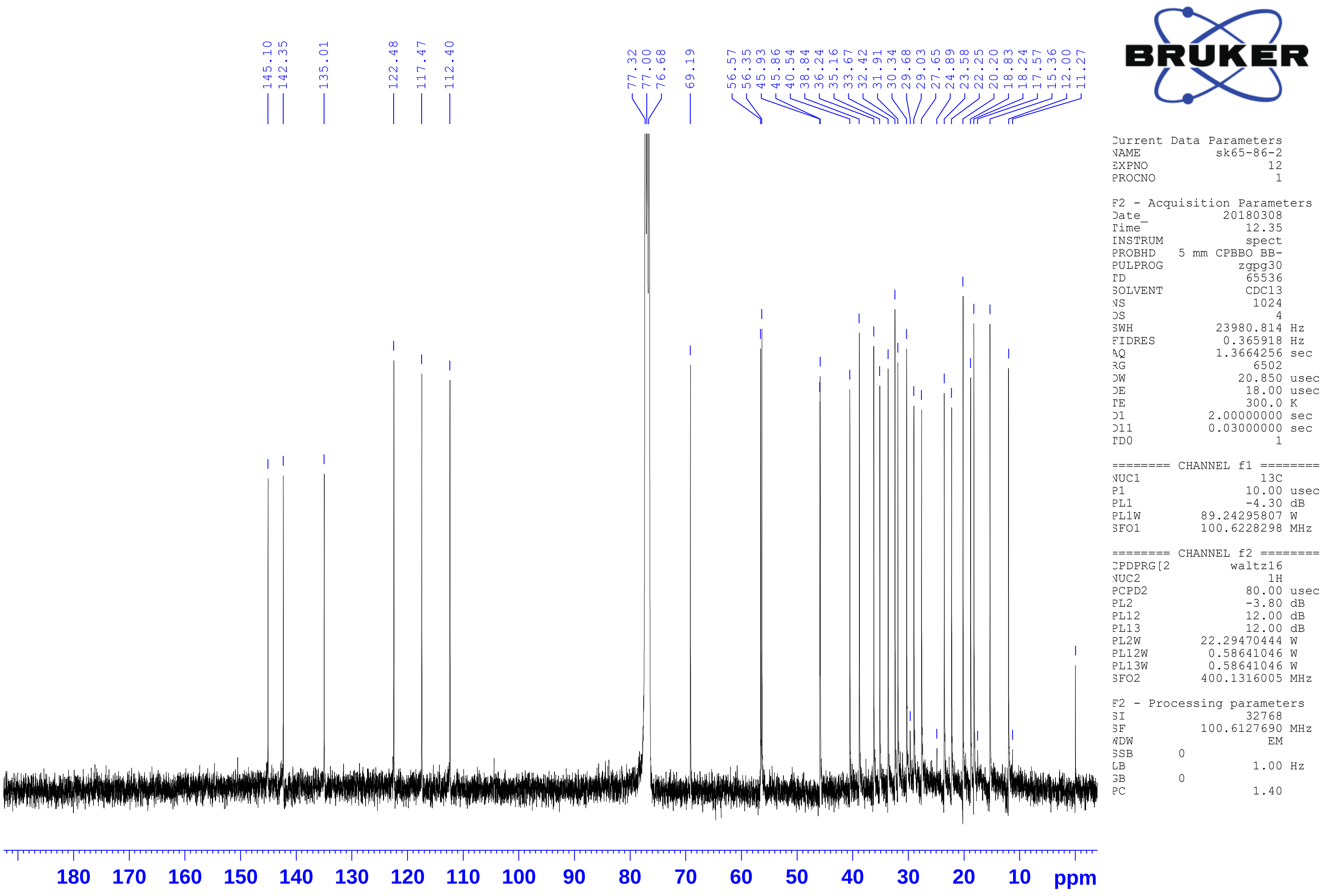

Figure S24. ^{13}C NMR spectrum of vitamin D_7 (**7**).

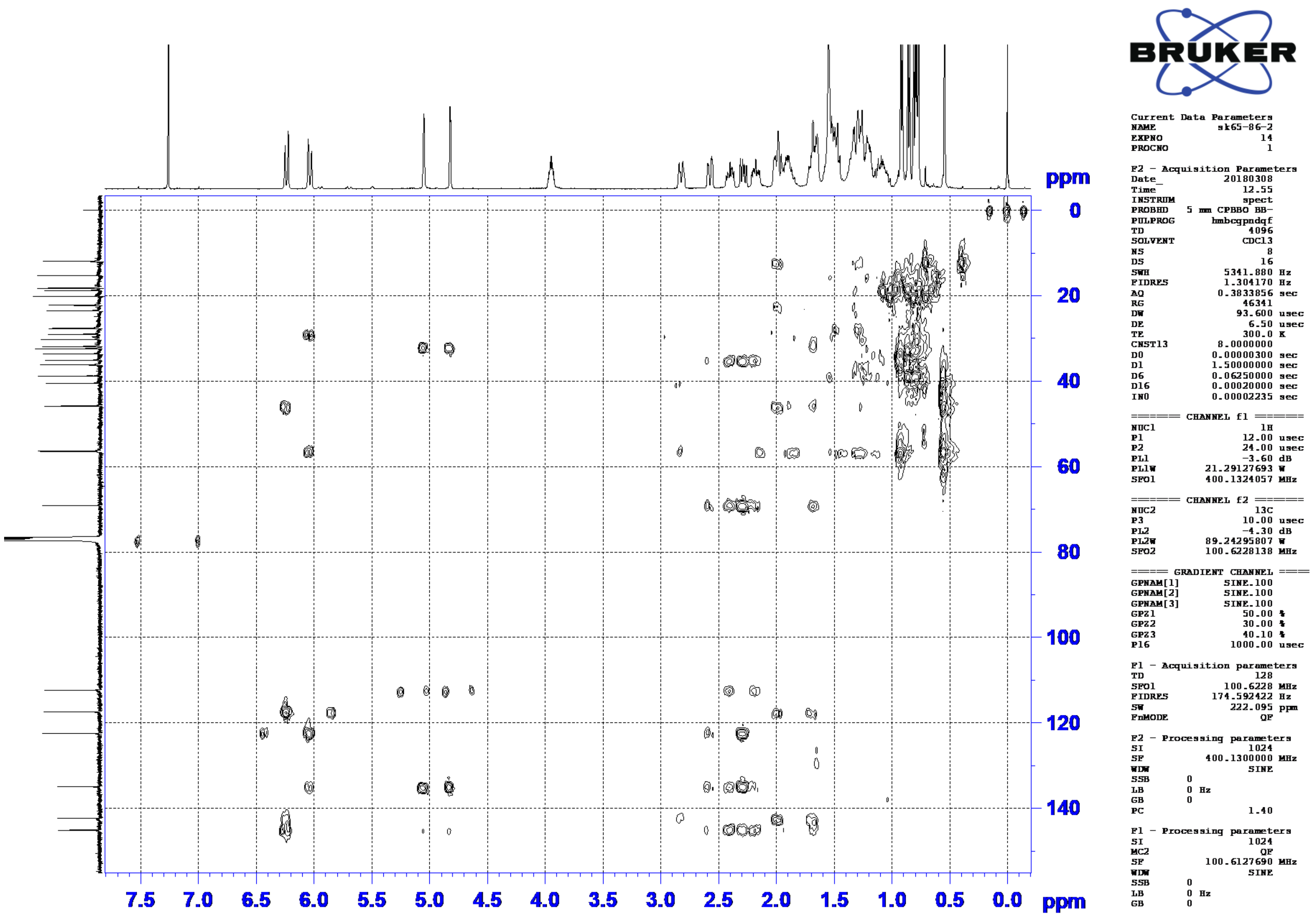

Figure S25. HMBC NMR spectrum of vitamin D_7 (**7**).
